# A case report: An unusual presentation of adenoid cystic carcinoma of the lacrimal gland

**DOI:** 10.1097/MD.0000000000033446

**Published:** 2023-03-31

**Authors:** Meiqin Liang, Zhirui Yu, Feng Wang

**Affiliations:** a Department of Ophthalmology, Changzhi People’s Hospital, Changzhi, China.

**Keywords:** adenoid cystic carcinoma, bone destruction, lacrimal gland

## Abstract

Adenoid cystic carcinoma (ACC) is the most common malignant epithelial tumor of the lacrimal gland with the highest malignant degree. ACC of the lacrimal gland is characterized by symptoms of <1 years duration. We present a 38-year-old male patient who complained of an enlarging mass in the left lacrimal fossa for almost 10 years previous to the diagnosis of ACC.

**Patient concerns:**

A 38-year-old male patient visited our ophthalmology clinic with a chief complaint of a mass in his left upper lid, which had enlarged significantly over the previous months.

**Diagnoses:**

Magnetic resonance imaging with intravenous Gadobutrol showed moderate and homogenous mass enhancement. Bone destruction is found. The periosteum is not eroded. The magnetic resonance imaging finding was supportive for malignancy. Histopathological examination of the specimen revealed solid tumor showing a cribriform pattern mixed small amount of basaloid cell proliferation. Therefore, the final diagnose was Adenoid cystic carcinoma of the lacrimal gland.

**Interventions:**

The treatment included en bloc resection of the mass and adjacent bone and radiotherapy.

**Outcomes:**

In 1 year follow-up after operation, there is no recurrence. Visual acuity is 30/30. The left eye shows limitation on abduction.

**Lessons:**

The present case demonstrates an unusual progression of ACC of the Lacrimal Gland.

## 1. Introduction

Adenoid cystic carcinoma (ACC) is an uncommon malignancy that arises in secretory glands, particularly the major and minor salivary glands. ACC of the lacrimal gland accounts for 1.6% of all orbital tumors.^[1]^ Lacrimal gland adenoid cystic carcinoma is an aggressive tumor with a tendency to invade intracranially. Radiography may help in the diagnosis of the lacrimal gland ACC. We report a patient with adenoid cystic carcinoma of the lacrimal gland in which the initial symptoms appeared 10 years previous to the diagnosis.

## 2. Case report

A 38-year-old male patient was admitted to our out-patient clinic of Changzhi People’s Hospital with a complaint of a mass in his left upper lid, which had enlarged significantly over the previous months. About 6 months before visiting our hospital, he started to perceive progressive proptosis of the left eye without the symptoms of pain, redness and impaired vision.

Ten years previously, the patient noticed a small painless mass in the temporal aspect of his left upper lid. However, the mass did not enlarge during the past 10 years.

**Figure 1.**
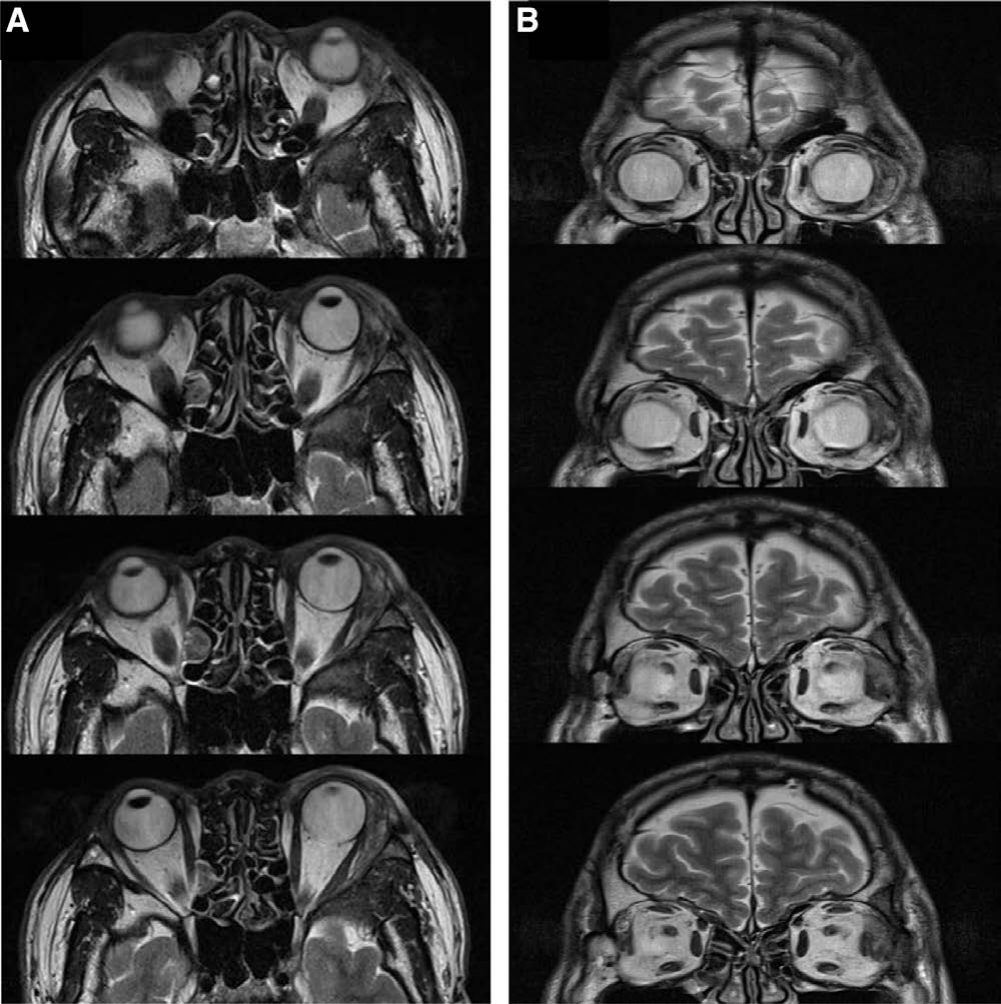
T2-weighted axial, (A) and coronal (B) magnetic resonance image showing left orbital mass in the area of the lacrimal gland (bony wall of orbit was infiltrated, the periosteum was intact).

**Figure 2.**
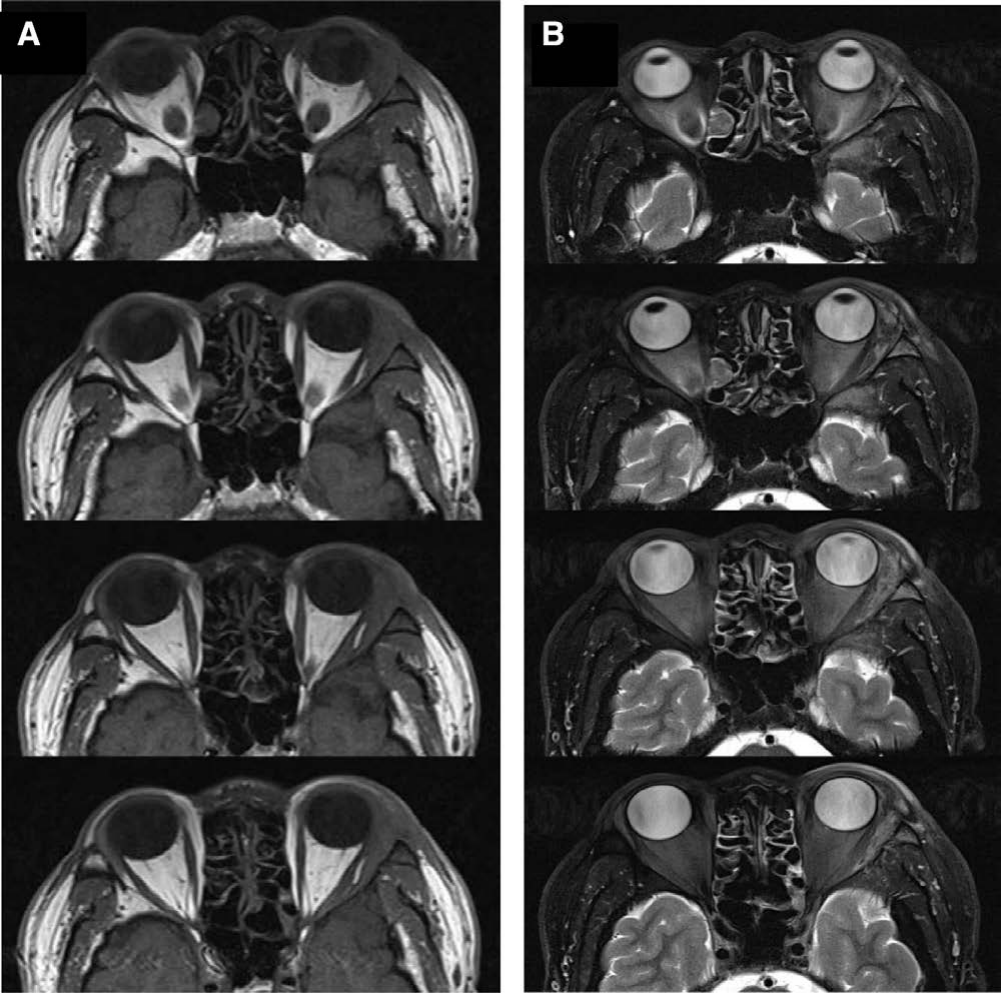
T1-weighted axial (A) and T2-fat suppressed axial (B) magnetic resonance image showing left orbital mass in the area of the lacrimal gland (bony wall of orbit was infiltrated, the periosteum was intact).

**Figure 3.**
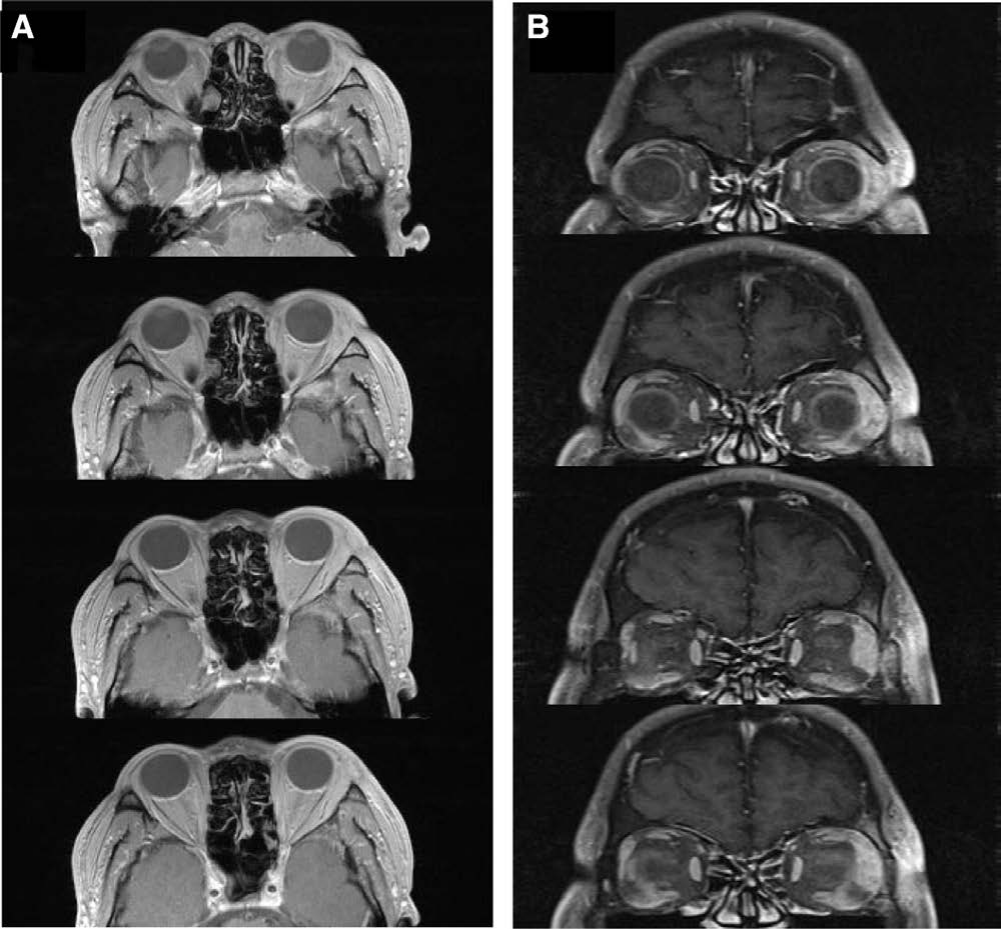
Axial (A) and coronal (B) gadobutrol-enhanced T1-weighted magnetic resonance image showing a left orbital mass in the area of the lacrimal gland (bony wall of orbit was infiltrated, the periosteum was intact).

On the examination in our hospital, visual acuity was 30/30 and the intraocular pressure was 14 mm Hg in the left eye. The anterior and posterior segments of the left eye was normal. The mass in the left upper lid that could be touched was spherical, with immobile and about 7mm in dimension. The orbital pressure was + 2. The finding of exophthalmometer was 13 mm to 15 mm, the distance of the orbit was 110 mm. The right eye was normal. Computed tomographic scans of the orbit-facial area demonstrated a huge solid mass lesion at the left orbital cavity. Magnetic resonance imaging with intravenous Gadobutrol showed moderate and homogenous mass enhancement. Bone destruction is found. The periosteum is not eroded. The magnetic resonance imaging (MRI) finding was supportive for malignancy (Figs 1–3).

The patient refused exenteration and was treated with a superolateral orbitotomy with complete excision, including resection of the lacrimal gland mass and removal of the orbital lateral wall, followed by radiotherapy.

**Figure 4.**
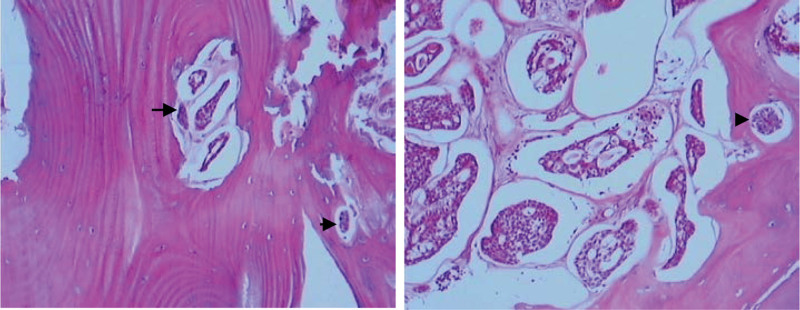
Histologic section of bony walls of orbit with tumor infiltration in (hematoxylin-eosin, ×100).

Histopathological examination of the specimen revealed solid tumor showing a cribriform pattern mixed small amount of basaloid cell proliferation (Fig. 4).

Postoperatively, the patient did well. Before he left hospital, Visual acuity was 20/30 and the left eye showed limitation on abduction. One month after surgery, he received radiotherapy because of a risk for the future recurrence of the tumor. In 1 year, follow-up after operation, there is no recurrence. Visual acuity is 30/30. The left eye shows limitation on abduction.

## 3. Discussion

Tumors of the lacrimal gland are uncommon in the clinical practice. They constitute approximately 25% of orbital tumors and are generally divided into 4 categories: inflammatory lesions, lymphomas, metastatic cancer, and epithelial tumors.^[2,3]^ Epithelial neoplasms represent approximately 20% to 50% of the lesions of the lacrimal gland. Adenoid cystic carcinoma is the most common malignant tumor of the lacrimal gland.^[4,5]^ Adenoid cystic carcinoma of the lacrimal gland has a dismal prognosis. Histologically, ACC are subtyped by their pattern of growth as tubular, cribriform, or solid.^[3,6]^ The solid variant has more aggressive biologic behavior and is associated with overall shorter survival.^[7]^

According to the literature, the adenoid cystic carcinoma of the lacrimal gland is characterized clinically by a painful palpable mass with rapid growth, proptosis and inferomedial displacement of the globe.^[8]^ Usually, the symptoms of growth and pain are briefer than 6 months. Growth history of a lesion in the lacrimal fossa for more than 6 months is highly suggestive of a benign tumor.^[9]^ With regard to symptoms, Wright et al^[10]^ Emphasized that pain is an important symptom of lacrimal gland malignancies because it implies perineural infiltration.

Our patient has a mass in his left upper lid without the symptoms of pain and redness for 10 years. He was operated on because of an acute enlargement of the mass. Histologic examination revealed an adenoid cystic carcinoma of the lacrimal gland. The superotemporal bony wall of orbit was infiltrated. However, the periosteum was intact.

MRI may be helpful in demonstrating along the border of the lesion. MRI may be poorer for detecting bony erosion. After our patient was conducted with intravenous gadobutrol, the signal intensity of the left superotemporal bony wall of orbit was not same to the right. Because of the presence of orbital bone infiltration, malignancy was considered in the diagnosis. However, the periosteum of the orbital bone was intact.

## 4. Conclusion

The present case demonstrates an unusual progression of adenoid cystic carcinoma of the Lacrimal Gland. This case suggests that adenoid cystic carcinoma should be considered in the diagnosis of an orbital tumor, even if benign is suspected based on duration of progressive mass and the symptoms without pain and redness.

## Author contributions

**Writing – original draft:** Meiqin Liang, Zhirui Yu, Feng Wang.
